# How stable are moral judgements? A longitudinal study of context dependency in attitudes towards patient responsibility

**DOI:** 10.1186/s12910-024-01035-x

**Published:** 2024-03-25

**Authors:** Berit H Bringedal, Karin Isaksson Rø

**Affiliations:** The Institute for Studies of the Medical Profession, PO Box 1152, Oslo, 0107 Norway

**Keywords:** Professional ethics, Priority setting, Personal responsibility, Physicians, Norway

## Abstract

**Background:**

Whether patients' life-style should involve lower priority for treatment is a controversial question in bioethics. Less is known about clinicians' views.

**Aim:**

To study how clinical doctors' attitudes to questions of patient responsibility and priority vary over time.

**Method:**

Surveys of doctors in Norway in 2008, 2014, 2021. Questionnaires included statements about patients' lifestyle's significance for priority to care, and vignettes of priority cases (only in 2014).

**Results:**

Attitudes were fairly stable between 2008 and 2021. 17%/14% agreed that patients' lifestyle should count, while 19%/22% agreed that it should involve lower priority to scarce organs. 42/44% agreed that smokers should have lower priority. Substantially more agreed in 2014. Regression analyses showed that being male, working in hospital, and younger age increased the likelihood of agreeing.

**Conclusion:**

A substantial minority of doctors agreed that lifestyle should be a priority criterion, possibly contrary to Norwegian legislation and professional ethics. The finding might be explained by the unspecified meaning of priority, increased scarcity-awareness, or socio-cultural trends towards individualism. The 2014 results indicate a framing effect; the vignettes may have primed the respondents towards accepting lifestyle as a criterion. We conclude that attitudes to normative questions are unstable and depend on context. A substantial minority of doctors seems to be positive to deprioritizing patients allegedly responsible for their illness. However, what deprioritization implies in practice is not clear.

**Supplementary Information:**

The online version contains supplementary material available at 10.1186/s12910-024-01035-x.

## Background

In healthcare, large resources are spent on diseases that – in principle—could have been avoided or reduced by life-style changes, or other behavioural changes [1]. Smoking, high intake of alcohol, unhealthy food and little physical activity are factors that contribute to the most common non-contagious diseases, like diabetes, cardiovascular disease, cancer, respiratory disease and depression. In Norway, half of the deaths in 2018 were due to cardiovascular disease and cancer, and a large proportion of treatment resources are spent on lifestyle-related diseases [2].

There is wide agreement that it is desirable to reduce the extent of lifestyle-related disease, but how this should be done is more controversial. How far can or should health authorities go in order to influence personal life-style choices? Further, the interrelatedness between health, lifestyle and socio-cultural and economic factors [3, 4] implies a risk of social bias and, in the worst case, increased social inequalities in health, since life style and socioeconomic status is closely connected.

There are ongoing debates in medical ethics on whether patients whose lifestyle increases the risk of disease should have lower priority than others to receiving healthcare. Should e.g. a patient with a congenital lung disease be preferred for treatment, compared to a patient with lung disease caused by smoking? The philosopher Ronald Dworkin introduced the view that there is a relevant moral distinction between "brute luck" and "option luck", where the former denotes an outcome over which an individual has no control, while the latter denotes an outcome that an individual can control through fully informed and deliberate choice of action [5]. The so called "luck egalitarianism" was born, and a number of scholars have contributed to developing the perspective [6–8]. Frequently, a distinction is commonly made between backward looking and forward looking responsibility. In a backward looking perspective, actions in the past are considered as a reason for allotting lower priority, e.g. the fact that the patient is a smoker. In a forward looking perspective, access to treatment is made dependent on the patient's future behaviour, e.g. in the form of a contract where s/he agrees to quit smoking [6].

Although much debated in the literature, there are relatively few empirical studies of clinical decision makers' thoughts about this. A Swedish experimental study of clinicians and the general public found that both groups were inclined to prioritise a non-smoker compared to a smoker for an expensive treatment [9]. A recent qualitative study of clinicians in Norway found that they were generally positive to considering patient responsibility in some cases, but reluctant towards establishing this as a formal priority criterion [10]. A survey comparing.g British and Norwegian clinicians found that up to 50% of the doctors, in both countries, considered an unhealthy lifestyle to be a reason for lower priority [11], while a previous Norwegian study found that 20–25% of them supported this view [12]. A review of empirical studies of bias towards patients with obesity found that healthcare providers hold strong negative attitudes about these patients, and that this affects their behaviour and decision making [13]. This might be so because the provider considers the patient to be responsible for his/her obesity, though this was not explicitly studied.

The doctor's own life style may also influence treatment and recommendations to patients with life style induced symptoms or disease [14]. This indicates that doctors own lifestyle could influence their moral considerations about patient responsibility.

The limited knowledge of how doctors think about the significance of patient responsibility is problematic since they are "street level bureaucrats" [15]. The term refers to the frontline workers, those who do the actual distribution of resources between clients, or patients. Their attitudes and thoughts are important to study because it provides data on relations between the official policy goals and the actual decisions.

Attitudes and moral considerations can differ between GPs and hospital doctors. In Norway, health care is publicly funded, with equal access to treatment, irrespective of financial capacity. Each resident is assigned to a named GP, and access to hospital care is dependent on a referral from a GP. An important part of the GP´s role is thus to take responsibility for follow up of patients, which differs from the hospital specialist´s role. This could involve different views on the question of patient responsibility.

As in comparable countries, Norwegian health care faces increasing resource constraints. When more attention is put on the fact that resources are limited, attitudes towards life style and patient responsibility may also become more topic.

A recent study of Norwegian policy documents on priority setting in health care concludes that patient responsibility "is repeatedly rejected as a necessary priority criterion", and that considerations of patient lifestyle is delegated to the bedside. This may "risk a grey zone between medicine and morality"[16].

Data about doctors' opinions about lifestyle and priority can be used to cultivate the profession's moral sensibility by stimulating discussion and moral awareness among those who allocate in practice. There is a need to heighten the ethical awareness among the professionals, precisely because of their role as street level bureaucrats.

In a survey, expressed attitudes are/can be shaped by the research design, as well as by external societal factors. If e.g. a particular normative issue was much discussed at the time, this will influence the responses. Furthermore, normative questions are potentially more prone to such contextual influences than other questions. We are less likely to hold stable normative views, since they normally involve dilemmas, where there are valid arguments in favour of opposite conclusions.

Our study will add to the relatively scant empirical literature on doctors' views on priority setting in healthcare. We focus on moral values, their volatility, and how they can be influenced by context and framing. Finally, our aim is to contribute to the medical profession's own discussions of the significance of patients' lifestyle in the distribution of scarce resources.

## Research questions

- Do doctors in Norway think that priority to healthcare should depend on the patient's alleged responsibility for the disease?
- Which factors should be considered when prioritizing on the basis of patient responsibility?
- How stable are their views over time?
- How do attitudes vary with age, gender and work place?
- (How) does context and framing impact on these attitudes?

## Material and methods

### Participants

The participants are members of a panel, a representative sample of 1500–2300 (depending on year of study) working doctors in Norway. The panel was established in 1994, biennially surveyed by questionnaires. Its representativity is judged through a comparison with the membership register in the Norwegian Medical Association, comprising more than 95% of the doctor population (see Table 1 for a comparison of the panel and the doctor population). Representativity is further ensured by younger doctors being admitted when doctors leave the panel for retirement or other reasons. The data in this study are from the 2008, 2014 and 2021 dispatches of the survey. 520 doctors responded at all three time points.

**Table 1:** Characteristics of the samples and the population^a^ of doctors in each year (in parenthesis). Gender and work position given in percentages, mean age in years

	2008	2014	2021
Female	37.1 (40.8)	38.3 (47)	52.8 (53.1)
GPs	25.6 (24.5)	23.7 (24.8)	20.6 (22.8)
Hospital doctors	55.5 (55)	57 (55.1)	59.2 (57)
Other positions	18.9 (20.5)	19.3 (20.1)	15.9 (21.2)
Mean age	48 (44)	55 (44)	45 (46)
N	1052–1056	1135–1139	1553–1556

^a^Source: Occupationally active doctors below 70 years of age, Statistics of The Norwegian Medical Association Yrkesaktive leger i Norge (legeforeningen.no)

### Longitudinal design

The question of stability of values can be studied on an individual as well as on a population level, In the first case, the question is whether an individual holds the same values over time, in the second whether a population does this. Our material contains one group of doctors who responded all three survey years, which will reflect individual stability, while the other responded one or two times. The latter can be considered to reflect the population of doctors' views on the question.

The questions on patient responsibility were phrased the exact same way all three years, with one exception: In 2014, three scenarios on patient responsibility preceded the questions that were asked all three years.

### Variables

Independent: Gender (dichotomic), age (continuous), and work position (hospital doctors, GPs and others).

**Fig. 1:**
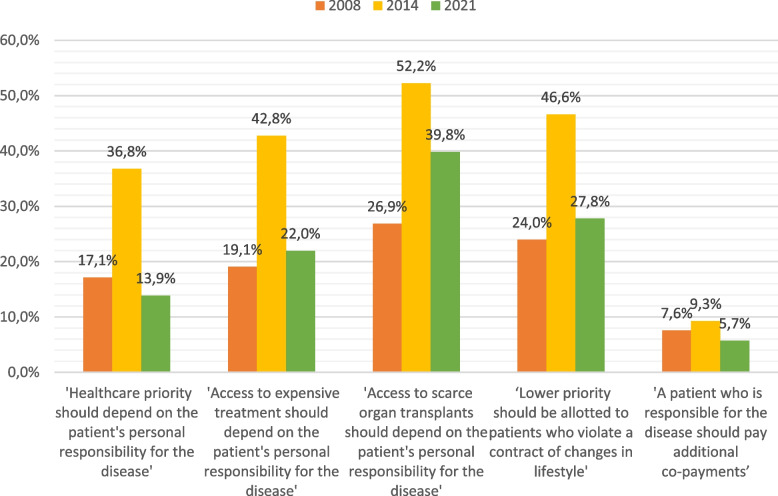
Proportions who partly or fully agree with statement

Dependent: Fig. 1 shows the first group of questions consisting of five statements about patient responsibility for the need for healthcare and whether this should impact on priority to healthcare. The response alternatives were on a scale graded from strongly disagree [1] to strongly agree [5].

**Fig. 2:**
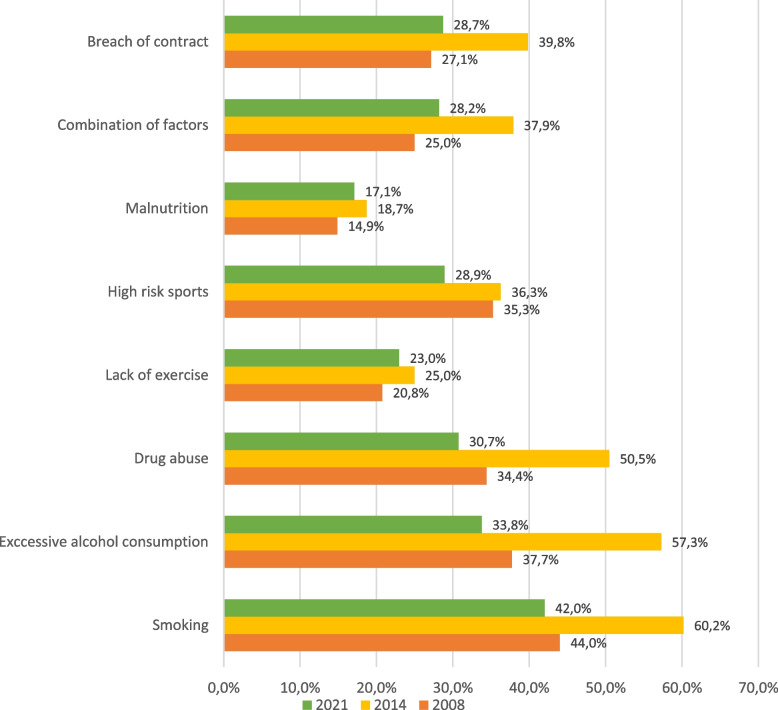
Factors to be taken into account when allocating healthcare

Figure 2 shows statements about specific lifestyle factors which could influence the patient's priority. Nine conditions were stated where the doctors should score "yes", "no" or "don't know". The questions from the survey in 2008 [3] were repeated in 2014 and in 2021.

The questions for this study constituted only a minor part of a larger questionnaire. The relevant excerpt is provided in Table F in the supplementary file.

### Statistical analyses

The results are first presented descriptively, as frequency distributions (percentages). To investigate whether the changes between survey year were significant, we performed chi-square tests. To investigate differences between subgroups of doctors, we used regression analyses. Although the dependent variable—agreement with the statements—is an ordinal scale variable, the response distributions were approximately normal. Hence, we applied multivariate linear regression analyses. SPSS 26 was used.

## Results

Response rates: 2008: 65% (1072 of 1649), 2014: 75% (1158 of 1545), 2021: 70% (1617 of 2316). Table 1 shows the distribution of gender, age and work positions compared to the general population of working doctors in Norway.

As expected, the number of female doctors increases over time. The proportions in the three work categories are relatively similar, though the relative number of hospital doctors increases, while the proportions of GPs and other positions decrease. These patterns reflect the population.

The 2014 sample deviates from the population in terms of age and gender. Mean age differed by 11 years, and the proportion of female doctors was also low compared to the population (38% compared to 47% in the population).

Figure 1 shows that the response pattern was fairly stable between 2008 and 2021, while in 2014, the proportion of doctors who almost or completely agreed that patient lifestyle should count was approximately doubled. The exception is the statement about co-payments, which is stable and low. (Table A in the Supplementary file provides the detailed responses, including the percentages who responded partly and completely (dis-) agreement, and neutral.)

Although the difference in responses in 2014 compared to the two other years is the most prominent result, there are statistically significant differences between 2008 and 2021 (Table C in the Supplementary file). There was a statistically significant reduction in the proportion of doctors who agreed with the general statement and the statement about co-payments. The largest change in agreement concerned access to scarce organ transplants, which increased from 35.2 to 50.9% ('neutral' excluded). The change in the proportion of doctors who responded 'yes' to the listed lifestyle factors was similar to the responses to the general statements, see Fig. 2. Here too 2014 is a persistent outlier, accounting for the highest proportion of yes-responses on all factors.

Among the participants who responded at all three time points (*n* = 520) the proportions who agreed partly or fully with the five statements did not differ significantly from the proportions listed in Fig. 1, measured by overlapping 95% confidence intervals. Only regarding access to scare organ transplants in 2021 the proportion was higher among doctors responding at all three points in time than among the whole group in 2021. Please see Appendix Table F for details.

Table B in the Appendix provides all response alternatives in this group of questions, and Table D shows that all significant changes between 2008 and 2021 were reductions in agreement.

### Variations between subgroups

To study differences between general practitioners and hospital doctors we performed regression analyses, controlling for gender and age. We dichotomised the job variable (GP or hospital), and excluded doctors in other positions. Since the distribution of the dependent variables were approximately normal, we used linear regression. The B-coefficients and associated significances are shown in Table 2, while the complete results of the regression analyses are provided in the supplementary file (Table E, i-v).

**Table 2:** The effect of work position on agreement with statement, controlled for age and gender. Linear regression analysis. *N* = 1052–1056 (2008), 1135–1139 (2014) and 1553–1556 (2021)

	**2008**			**2014**			**2021**		
**Statement**	**Age**	**Gender**	**Job**	**Age**	**Gender**	**Job**	**Age**	**Gender**	**Job**
	B	B	B	B	B	B	B	B	B
	(sig)	(sig)	(sig)	(sig)	(sig)	(sig)	(sig)	(sig)	(sig)
'Healthcare priority should depend on the patient's personal responsibility for the disease'	.006	.076	.03	-.005	-.044	.019	-.01	-.243	.183
	(.097)	(.32)	(.699)	(.16)	(.566)	(.821)	(< .001)^b^	(< .001)^b^	(.005)^b^
'Access to expensive treatment should depend on the patient's personal responsibility for the disease'	0	-.177	-.02	.006	.076	.03	-.012	-.193	.078
	(.949)	(.029)^a^	(.808)	(.097)	(.32)	(.699)	(< .001)^b^	(.003)^b^	(.289)
'Access to scarce organ transplants should depend on the patient's personal responsibility for the disease'	-.01	-.157	-.047	.006	.076	.03	-.026	-.187	-.09
	(.017)^a^	(.07)	(.592)	(.097)	(.32)	(.699)	(< .001)^b^	(.009)^b^	(.263)
‘Lower priority should be allotted to patients who violate a contract of changes in lifestyle'	-.003	-.088	-.172	.006	.076	.03	-.011	-.197	-.272
	(.408)	(.272)	(.034)^b^	(.097)	(.32)	(.699)	(< .001)^b^	(.003)^b^	(< .001)^b^
'A patient who is responsible for the disease should pay additional co‐payments’	-.01	-.187	-.039	.006	.076	.03	-.007	-.052	-.049
	(.002)^b^	(.01)^a^	(.591)	(.097)	(.32)	(.699)	(.003)^b^	(.345)	(.424)

Response alternatives statements: 1 Disagree fully, 2 Disagree partly, 3 Neutral, 4 Agree partly, 5: Agree fully,

Age Years, Gender Male 0, Female 1, Job Hospital 0, GP 1

^a^95% level significance

^b^99% level significance

Table 2 shows that there were no significant effects of the independent variables on degree of agreement in 2014. In 2008, male doctors were more likely to agree with the statement about expensive treatment and transplant, when controlling for job and age. This year, increasing age correlated with a reduction in the likelihood of agreeing with the statement on transplants, and fewer GPs agreed that violating a contract of changes in lifestyle should lead to lower priority.

In 2021, the responses followed a similar pattern for four of the five statements: Being male, of younger age, and working in a hospital, was associated with being more positive to letting patient lifestyle influence on priority for healthcare.

## Discussion

A large minority of doctors agreed that patient lifestyle should play a role in allocation of healthcare resources. This is surprising, since this can be interpreted to be contrary to Norwegian legislation on priority setting, as well as to professional ethics. Another surprising finding was the 'outlier year' 2014; why did almost twice as many doctors consider patient responsibility as a priority criterion this year compared to 2008 and 2021?

It should be noted that we did not provide a precise definition of "prioritisation" in the questionnaire. The doctors might have interpreted the concept to mean that patients with alleged personal responsibility are put further down in a queue, involving increased waiting time. Alternatively, it could involve to be denied treatment altogether, especially in situations with grave scarcity, as with scarce organ transplants. Finally, it could be reasonable to interpret lower priority as receiving less help than others, alternatively a different kind of help. In this case, less resource use/lower costs, or increased co-payments might be the result, or that these patients are in need of entirely different kinds of help (whether requiring less resources or not).

Although we do not know exactly what the informants had in mind with "lower priority", we nevertheless have data indicating that the attitudes vary significantly over time (and the doctors who answered at all three time points did not differ from those who answered at one or two occasions), as well as between subgroups of doctors. We interpret these variations as the result of *context dependency*, meaning that attitudes are influenced by the society, or societal changes, the contents of the clinical work/the clinical setting, and/or the framing of the questions in the questionnaire.

### Societal factors

In the last decades, stake holders in healthcare have become increasingly aware that resources are limited. In Norway, Norwegian Official Reports, White Papers and guidelines for priority setting in healthcare have been issued since 1987 [18, 19], the establishment of publicly funded health economic institutions, and the recent Choosing Wisely campaigns have all contributed to broad acknowledgement of resource scarcity. Today, the cost of treatment is considered a legitimate consideration – in addition to treatment effect and severity of disease. How resources best should be distributed between patients or interventions has thus increasingly become a legitimate question, also within the healthcare system and among health professionals. As a result, the discussions about priority-setting have changed, in Norway as well as internationally. There is gradually less support for the view that ranking of interventions and patients is unethical. Instead, the most widespread view is now the opposite, namely that it is unethical *not* to rank-order in accordance with effect and severity [20].

Another possible explanation of the deviant 2014-results could be that something happened in Norway in the period before the survey, events that reasonably could have influenced the responses. Apart from the ongoing gradual attention to budget constraints and the necessity of prioritisation, we are not able to pinpoint any specific events that could affect this year's responses in particular. The 2014 Norwegian Official Report on priority setting in health care [21]– which involved extra attention to prioritisation in health care—was published after the survey was conducted.

### Clinical setting

Compared to hospital doctors, GPs were less inclined to let patient responsibility count in decisions on priority. This can be a result of the different roles of GPs and hospital doctors.

Generally, doctors have at least four, sometimes inconsistent, roles [22]: Gatekeeper or administrator, patient advocate, professional, and private individual. The gatekeeper/administrator is expected to act in accordance with laws and system requirements, as well as taking responsibility for population health and fair distribution of resources. The professional must adhere to good practice and professional ethics. The patient's advocate must ensure that care is in line with the patient’s views and interests. Finally, as a private individual, the doctor would not want to act contrary to personal core values and interests. The emphasis between roles may vary between positions in the healthcare system.

Compared to hospital specialists, GPs have more contact with, ergo more information about, their patients. This may lead to acknowledging the complexity of causes behind health and disease [17], which in turn can lead to being less categorical about the individual's personal responsibility for the disease. As a result, fewer GPs will agree that patient responsibility should guide the priority between patients.

Still, many GPs agreed to consider patient responsibility in a priority setting. That "lower priority" was undefined introduces a possible nuancing, "lower priority" could mean "another treatment". Rather than denying healthcare, some respondents may have considered that the patient's condition required alternative care. However, it should not be ignored that a substantial minority of doctors, hospital doctors as well as GPs, considered it right to deprioritize on the basis of lifestyle.

GPs have a gatekeeper role. Theirs is the responsibility to decide whether a patient should be referred to specialist healthcare, or be treated in, the less costly, primary care. There is an expectation that the GPs are cost conscious in the sense that they avoid unnecessary referrals, which may also lead to, more or less justified, strict decisions.

### Framing effects in the questionnaire

In contrast to the questionnaires in 2008 and 2021, the 2014 dispatch included three vignettes, each describing a concrete priority situation, presented in detail in another paper [11]. Briefly, the vignettes describe three situations where a doctor is asked to choose one of two patients, both of them in the same, possibly fatal, health state. In one case, both patients are in need of a lung transplant due to a lung disease, the only difference between them is the cause of their disease. One of the patients has a congenital disease causing the need for the treatment, the other is a smoker. The vignettes preceded the same questions as was surveyed in 2008 and 2021.

Albeit the vignettes' hypothetical and 'dramatic' character, it is possible that they primed the respondents into being more open to the view that patient lifestyle should be considered in "tragic choices" [23] specifically, and in prioritisation in healthcare in general.

The comparative study of doctors in Norway and the United Kingdom [11] included the vignettes. The substantially higher number of doctors who agreed that patient lifestyle should be considered may have been caused by this priming. It would have been interesting to see if a study of British doctors without the vignettes had shown the same results as the latest Norwegian survey.

Framing of questions are shown to influence responses in surveys. An experimental survey of public attitudes towards assisted dying showed that responses were dependent on how the question was phrased [24]. Björk et al. found that exchanging the patient from smoker to non-smoker changed the respondents' view on whether a patient should receive an expensive and marginally life- prolonging treatment [9].

### What does "responsibility" imply?

A major part of the discussions on patient responsibility in general, and luck egalitarianism in particular, concerns where to draw the 'responsibility line'. Is substance dependency, like smoking, under the individual's control similarly as other life style factors, like physical activity or food habits? The difficulty in determining the limits of responsibility introduces the complex philosophical question of free will [4], the influence of socioeconomic factors and the risk of enhanced social inequality, and the difficulties in establishing practical priority principles capable of combining patient responsibility while avoiding adverse consequences [25].

### Unjustified moralism?

Neither Norwegian legislation [26] nor professional ethics [27] include patient lifestyle or behaviour as a legitimate criterion for decisions on priority to care. The Geneva Declaration, "The physician's Oath" [28] states that no other concern than the patient's medical need should count in a decision about healthcare. Individual characteristics leading to discrimination based on gender, social status or ethnicity are explicitly mentioned as illegitimate concerns – while lifestyle is not. Still, it is reasonable to assume that the intention of the code is to express that the reasons for the need of healthcare are irrelevant for a decision about priority for treatment.

Norwegian law [26] states three criteria that should be considered: potential benefit of treatment, cost of treatment, and severity of the disease. A patient's lifestyle or behaviour is not a priority criterion. However, guidelines recommend to prioritize according to the health effect of treatment (priority increases when benefit increases, other things equal), which may involve lower priority to measures where lifestyle factors can reduce the effect of the measure.

Although we are aware that there are several legitimate interpretations of the priority question in this study, as well as in clinical practice, we cannot rule out that some of the doctors find it justified to give lower priority to some patients on the basis of their alleged responsibility alone. Some unacknowledged bias and stereotyping exist in healthcare as well as in other parts of the society [13, 29], involving that there is a need of explicating and discussing questions about patient lifestyle, individual responsibility, priority setting, and what kind of care the patient needs.

Furthermore, health risk behaviour follows a social pattern. There are far more people who smoke, are sedate, and/or have a high BMI among people with low socio-economic status. The large social inequality in health in Norway, as in most other countries, can be attributed to different lifestyles [14, 15]. If patients are downgraded on the basis of "responsibility", that the individual can be personally blamed for her ill health, there is a great risk that social inequality will increase.

It is our hope that this study can initiate discussions among the doctors themselves, thus contributing to cultivating moral values. However, in addition to the cultivation of moral values, more knowledge about the complex relations between socioeconomic status, culture, life style and health is needed. The Whitehall Studies showed that less than half of the social gradient in health was directly attributable to classical biomedical risk factors [3]. Doctors, as well as other decision makers in the health care system, should acknowledge this in efforts to improve patient and population health.

### Strengths and weaknesses

One strength was the longitudinal design, which makes it possible to study how the same variable varies over time. Another is that the survey data was followed by a qualitative focus group interview.

The influence on the results from the research design itself is always a challenge. This is not only demonstrated in this study, but was also included as one of the research questions.

A weakness is that beliefs and attitudes can be hard to detect from what people say, in contrast to how they behave. Self-presentation involves biases like the wish to accommodate the researcher, or to present oneself in a flattering light. Hence, despite the substantial minority who agreed with the statements, we do not know its implications in clinical practice.

## Conclusion

A substantial minority of doctors agreed that lifestyle should be a priority criterion, contrary to Norwegian legislation and professional ethics. Our interpretation of the result is threefold: Increased scarcity awareness may have led to increased attention to the patients' personal responsibility. "Lower priority" can have been interpreted to mean other kinds of care. The study design may have primed the respondents to agreeing with the statements. Three lessons are drawn: Attitudes to normative questions are unstable and depend on context. The practical implication of "lower priority" should be explicit. A substantial minority of doctors are positive to deprioritizing patients allegedly responsible for their illness. How this view would unfold in practice is, however, unclear.

### Supplementary Information

**Supplementary Material 1.**

## Data Availability

The datasets generated and/or analysed during the current study are available from the corresponding author on reasonable request.
