# Supplementary material for: How stable are moral judgements? A longitudinal study of context dependency in attitudes towards patient responsibility
Source: BMC Med Ethics. 2024 Mar 25;25:36. doi: 10.1186/s12910-024-01035-x (PMC10962157; doi:10.1186/s12910-024-01035-x)
Supplement: Supplementary file 1 — Supplementary Material 1. [file 12910_2024_1035_MOESM1_ESM.docx]

***Bringedal and Isaksson Rø: How stable are moral judgements?***

***Supplementary file***

*Table A Agreement with statements 2008 and 2021. All response alternatives. Percentages.*

|  | Should depend generally | | | High cost treatment | | | Scarce organ transplants | | | Breach of contract | | | Co-payments | | |
| --- | --- | --- | --- | --- | --- | --- | --- | --- | --- | --- | --- | --- | --- | --- | --- |
|  | **2008** | **2014** | **2021** | **2008** | **2014** | **2021** | **2008** | **2014** | **2021** | **2008** | **2014** | **2021** | **2008** | **2014** | **2021** |
| Disagree completely | 24.1 | 12.1 | 22.2 | 23.0 | 10.4 | 21.4 | 20.8 | 8.7 | 15.2 | 18.4 | 7.5 | 16.2 | 43.0 | 37.3 | 47.9 |
| Disagree partly | 33 | 26.4 | 43.6 | 32.6 | 24.1 | 37.2 | 28.6 | 18.6 | 26.1 | 31.3 | 17.2 | 31.2 | 30.9 | 30.5 | 31.8 |
| Neutral | 25.9 | 24.7 | 20.2 | 25.3 | 22.7 | 19.4 | 23.8 | 20.4 | 18.9 | 26.3 | 28.7 | 24.8 | 18.6 | 23.0 | 17.6 |
| Agree partly | 15.9 | 33.8 | 13.1 | 16.2 | 37.7 | 19.6 | 22.7 | 44.2 | 29.9 | 21.1 | 42.1 | 24.1 | 6.5 | 8.1 | 6.4 |
| Agree completely | 1.2 | 3.0 | 0.8 | 2.8 | 5.1 | 2.3 | 4.2 | 8.0 | 9.9 | 2.9 | 4.5 | 3.7 | 1.0 | 1.1 | 1.0 |

*Table B Which factors should count? All response alternatives 2008, 2014, 2021. Percentages.*

|  | 2008 | | | 2014 | | | 2021 | | |
| --- | --- | --- | --- | --- | --- | --- | --- | --- | --- |
|  | Yes | No | Don't know | Yes | No | Don't know | Yes | No | Don't know |
| Overweight/obesity | 26.7 | 56.9 | 16.3 | 36.6 | 45.1 | 18.3 | 24.3 | 63.1 | 12.6 |
| Smoking | 44.0 | 45.1 | 10.9 | 60.2 | 28.5 | 11.3 | 42.0 | 47.6 | 10.4 |
| Excessive alcohol consumption | 37.7 | 48.5 | 13.8 | 57.3 | 29.1 | 13.6 | 33.8 | 53.5 | 12.7 |
| Drug abuse | 34.4 | 50.9 | 14.7 | 50.5 | 33.3 | 16.2 | 30.7 | 56.5 | 12.7 |
| Lack of exercise | 20.8 | 62.8 | 16.4 | 25.0 | 57.4 | 17.7 | 23.0 | 64.3 | 12.7 |
| High risk sports | 35.3 | 48.5 | 16.2 | 36.3 | 44.2 | 19.5 | 28.9 | 57.3 | 13.8 |
| Malnutriution | 14.9 | 70.2 | 14.9 | 18.7 | 61.8 | 19.4 | 17.1 | 67.2 | 15.8 |
| Combination of factors | 25.0 | 53.4 | 21.6 | 37.9 | 37.5 | 19.4 | 28.2 | 52.8 | 19.0 |
| Breach of contract | 27.1 | 55.0 | 17.9 | 39.8 | 34.0 | 26.1 | 28.7 | 51.4 | 19.9 |

*Table C Changes 2008-2021 Agreement with statements. Percentages.
 Differences tested with chi-square.*

| **'Healthcare priority should depend on the patient's personal responsibility for the disease'** | | | |
| --- | --- | --- | --- |
|  | 2008 | 2021 | Sig |
| Disagree | 76,9 | 82,6 | 0.002 |
| Agree | 23,1 | 17,4 |  |
| **'Access to expensive treatment should depend on the patient's personal responsibility for the disease'** | | | |
|  | 2008 | 2021 | Sig |
| Disagree | 74,5 | 72,7 | 0,393 |
| Agree | 25,5 | 27,3 |  |
| **'Access to scarce organ transplants should depend on the patient's personal responsibility for the disease'** | | | |
|  | 2008 | 2021 | Sig |
| Disagree | 64,8 | 50,9 | <0.001 |
| Agree | 35,2 | 49,1 |  |
| **‘Lower priority should be allotted to patients who violate a contract of changes in lifestyle'** | | | |
|  | 2008 | 2021 | Sig |
| Disagree | 67,5 | 63 | 0,045 |
| Agree | 32,5 | 37 |  |
| **'A patient who is responsible for the disease should pay additional co‐payments’** | | | |
|  | 2008 | 2021 | Sig |
| Disagree | 90,7 | 93,4 | 0,019 |
| Agree | 9,3 | 6,6 |  |

*Table D Changes 2008-2021 regarding factors that should count (percentages responding "Yes"). Differences tested with chi-square.
Significance: *0.05, **<0.001*

|  | 2008 | 2021 | Sig. | Trend |
| --- | --- | --- | --- | --- |
| Overweight/obesity | 31.9 | 27.8 | 0.035* | ↘ |
| Smoking | 49.4 | 46.9 | 0.240 |  |
| Excessive alcohol intake | 43.8 | 38.7 | 0.016* | ↘ |
| Drug abuse | 40.3 | 35.2 | 0.015* | ↘ |
| Lack of exercise | 24.8 | 26.3 | 0.430 |  |
| High risk sports | 42.1 | 33.6 | <0.001** | ↘ |
| Malnutrition | 17.5 | 19.9 | 0.163 |  |
| Combination of factors | 31.8 | 34.8 | 0.163 |  |
| Breach of contract | 33.1 | 35.8 | 0.163 |  |

*Table E, i)-v) Multinominal linear regression analyses*

| **Table E, i. Dependent Variable: Should depend on patient responsibility** | | | | | | | | | |
| --- | --- | --- | --- | --- | --- | --- | --- | --- | --- |
| survey_year | Model | | Unstandardized Coefficients | | Standardized Coefficients | t | Sig. | 95,0% Confidence Interval for B | |
|  |  |  | B | Std. Error | Beta |  |  | Lower Bound | Upper Bound |
| 2008 | 1 | (Constant) | 2,115 | 0,178 |  | 11,869 | <,001 | 1,765 | 2,465 |
|  |  | Alder | 0,006 | 0,004 | 0,06 | 1,662 | 0,097 | -0,001 | 0,013 |
|  |  | Gender | -0,076 | 0,077 | -0,036 | -0,996 | 0,32 | -0,226 | 0,074 |
|  |  | Jobb | 0,03 | 0,078 | 0,013 | 0,387 | 0,699 | -0,122 | 0,182 |
| 2014 | 1 | (Constant) | 3,141 | 0,181 |  | 17,326 | <,001 | 2,785 | 3,497 |
|  |  | Alder | -0,005 | 0,003 | -0,05 | -1,405 | 0,16 | -0,012 | 0,002 |
|  |  | Gender | -0,044 | 0,077 | -0,02 | -0,575 | 0,566 | -0,194 | 0,106 |
|  |  | Jobb | 0,019 | 0,082 | 0,008 | 0,227 | 0,821 | -0,142 | 0,179 |
| 2021 | 1 | (Constant) | 2,816 | 0,122 |  | 23,111 | <,001 | 2,577 | 3,055 |
|  |  | Alder | -0,01 | 0,002 | -0,127 | -4,297 | <,001 | -0,015 | -0,006 |
|  |  | Gender | -0,243 | 0,058 | -0,124 | -4,206 | <,001 | -0,356 | -0,129 |
|  |  | Jobb | 0,183 | 0,065 | 0,081 | 2,814 | 0,005 | 0,055 | 0,31 |
|  |  |  |  |  |  |  |  |  |  |
|  |  |  |  |  |  |  |  |  |  |
| **Table E, ii. Dependent Variable: High cost treatment** | | | | | | | | | |
| survey_year | Model | | Unstandardized Coefficients | | Standardized Coefficients | t | Sig. | 95,0% Confidence Interval for B | |
|  |  |  | B | Std. Error | Beta |  |  | Lower Bound | Upper Bound |
| 2008 | 1 | (Constant) | 2,521 | 0,188 |  | 13,418 | <,001 | 2,152 | 2,889 |
|  |  | Alder | 0 | 0,004 | -0,002 | -0,064 | 0,949 | -0,008 | 0,007 |
|  |  | Gender | -0,177 | 0,081 | -0,078 | -2,192 | 0,029 | -0,335 | -0,018 |
|  |  | Jobb | -0,02 | 0,082 | -0,008 | -0,243 | 0,808 | -0,18 | 0,141 |
| 2014 | 1 | (Constant) | 3,403 | 0,184 |  | 18,47 | <,001 | 3,041 | 3,765 |
|  |  | Alder | -0,006 | 0,004 | -0,059 | -1,658 | 0,098 | -0,013 | 0,001 |
|  |  | Gender | -0,164 | 0,078 | -0,074 | -2,102 | 0,036 | -0,317 | -0,011 |
|  |  | Jobb | -0,025 | 0,083 | -0,01 | -0,3 | 0,764 | -0,188 | 0,138 |
| 2021 | 1 | (Constant) | 3,048 | 0,138 |  | 22,161 | <,001 | 2,778 | 3,318 |
|  |  | Alder | -0,012 | 0,003 | -0,127 | -4,263 | <,001 | -0,017 | -0,006 |
|  |  | Gender | -0,193 | 0,065 | -0,088 | -2,96 | 0,003 | -0,321 | -0,065 |
|  |  | Jobb | 0,078 | 0,073 | 0,031 | 1,061 | 0,289 | -0,066 | 0,221 |
|  |  |  |  |  |  |  |  |  |  |

|  |  |  |  |  |  |  |  |  |  |
| --- | --- | --- | --- | --- | --- | --- | --- | --- | --- |
| **Table E, iii. Dependent Variable: Transplants** | | | | | | | | | |
| survey_year | Model | | Unstandardized Coefficients | | Standardized Coefficients | t | Sig. | 95,0% Confidence Interval for B | |
|  |  |  | B | Std. Error | Beta |  |  | Lower Bound | Upper Bound |
| 2008 | 1 | (Constant) | 3,143 | 0,201 |  | 15,622 | <,001 | 2,748 | 3,538 |
|  |  | Alder | -0,01 | 0,004 | -0,087 | -2,399 | 0,017 | -0,017 | -0,002 |
|  |  | Gender | -0,157 | 0,086 | -0,065 | -1,812 | 0,07 | -0,326 | 0,013 |
|  |  | Jobb | -0,047 | 0,088 | -0,019 | -0,535 | 0,592 | -0,219 | 0,125 |
| 2014 | 1 | (Constant) | 3,919 | 0,183 |  | 21,464 | <,001 | 3,561 | 4,278 |
|  |  | Alder | -0,012 | 0,004 | -0,121 | -3,4 | <,001 | -0,019 | -0,005 |
|  |  | Gender | -0,132 | 0,077 | -0,06 | -1,711 | 0,087 | -0,283 | 0,019 |
|  |  | Jobb | -0,014 | 0,082 | -0,006 | -0,17 | 0,865 | -0,176 | 0,148 |
| 2021 | 1 | (Constant) | 4,23 | 0,152 |  | 27,915 | <,001 | 3,933 | 4,527 |
|  |  | Alder | -0,026 | 0,003 | -0,25 | -8,581 | <,001 | -0,032 | -0,02 |
|  |  | Gender | -0,187 | 0,072 | -0,076 | -2,604 | 0,009 | -0,328 | -0,046 |
|  |  | Jobb | -0,09 | 0,081 | -0,032 | -1,119 | 0,263 | -0,248 | 0,068 |
|  |  |  |  |  |  |  |  |  |  |
|  |  |  |  |  |  |  |  |  |  |
| **Table E, iv. Dependent Variable: Breach of contract** | | | | | | | | | |
| survey_year | Model | | Unstandardized Coefficients | | Standardized Coefficients | t | Sig. | 95,0% Confidence Interval for B | |
|  |  |  | B | Std. Error | Beta |  |  | Lower Bound | Upper Bound |
| 2008 | 1 | (Constant) | 2,832 | 0,187 |  | 15,126 | <,001 | 2,464 | 3,199 |
|  |  | Alder | -0,003 | 0,004 | -0,03 | -0,827 | 0,408 | -0,01 | 0,004 |
|  |  | Gender | -0,088 | 0,08 | -0,039 | -1,098 | 0,272 | -0,246 | 0,07 |
|  |  | Jobb | -0,172 | 0,081 | -0,074 | -2,118 | 0,034 | -0,332 | -0,013 |
| 2014 | 1 | (Constant) | 3,653 | 0,168 |  | 21,777 | <,001 | 3,324 | 3,982 |
|  |  | Alder | -0,006 | 0,003 | -0,07 | -1,969 | 0,049 | -0,013 | 0 |
|  |  | Gender | -0,186 | 0,071 | -0,092 | -2,623 | 0,009 | -0,325 | -0,047 |
|  |  | Jobb | -0,243 | 0,076 | -0,11 | -3,211 | 0,001 | -0,391 | -0,094 |
| 2021 | 1 | (Constant) | 3,388 | 0,14 |  | 24,231 | <,001 | 3,114 | 3,662 |
|  |  | Alder | -0,011 | 0,003 | -0,122 | -4,134 | <,001 | -0,017 | -0,006 |
|  |  | Gender | -0,197 | 0,066 | -0,088 | -2,973 | 0,003 | -0,327 | -0,067 |
|  |  | Jobb | -0,272 | 0,074 | -0,105 | -3,652 | <,001 | -0,417 | -0,126 |
|  |  |  |  |  |  |  |  |  |  |

|  |  |  |  |  |  |  |  |  |  |
| --- | --- | --- | --- | --- | --- | --- | --- | --- | --- |
| **Table E, v. Dependent Variable: Co-payments** | | | | | | | | | |
| survey_year | Model | | Unstandardized Coefficients | | Standardized Coefficients | t | Sig. | 95,0% Confidence Interval for B | |
|  |  |  | B | Std. Error | Beta |  |  | Lower Bound | Upper Bound |
| 2008 | 1 | (Constant) | 2,496 | 0,168 |  | 14,817 | <,001 | 2,165 | 2,827 |
|  |  | Alder | -0,01 | 0,003 | -0,112 | -3,128 | 0,002 | -0,017 | -0,004 |
|  |  | Gender | -0,187 | 0,072 | -0,092 | -2,588 | 0,01 | -0,329 | -0,045 |
|  |  | Jobb | -0,039 | 0,073 | -0,019 | -0,538 | 0,591 | -0,183 | 0,104 |
| 2014 | 1 | (Constant) | 2,405 | 0,172 |  | 14,016 | <,001 | 2,069 | 2,742 |
|  |  | Alder | -0,006 | 0,003 | -0,064 | -1,785 | 0,075 | -0,012 | 0,001 |
|  |  | Gender | -0,087 | 0,073 | -0,042 | -1,202 | 0,23 | -0,23 | 0,055 |
|  |  | Jobb | -0,065 | 0,078 | -0,029 | -0,841 | 0,401 | -0,217 | 0,087 |
| 2021 | 1 | (Constant) | 2,128 | 0,116 |  | 18,33 | <,001 | 1,9 | 2,355 |
|  |  | Alder | -0,007 | 0,002 | -0,089 | -2,98 | 0,003 | -0,011 | -0,002 |
|  |  | Gender | -0,052 | 0,055 | -0,028 | -0,945 | F0,345 | -0,16 | 0,056 |
|  |  | Jobb | -0,049 | 0,062 | -0,023 | -0,8 | 0,424 | -0,171 | 0,072 |

***Table F Proportions who fully or partly agreed with statement among those who answered at all three time points versus all***

|  |  | **Doctors responding at all three points in time**  **N=520** | | **All respondents** | |  |
| --- | --- | --- | --- | --- | --- | --- |
| **Statement** | **Year** | **Proportion partly or fully agreeing with the statement** | **95% Confidence interval** | **Proportion partly or fully agreeing with the statement** | **95% Confidence interval** | **Significant difference** |
| Healthcare priority should depend on patient´s personal responsibility for the disease | 2008 | 15,2 | 12,1-18,3 | 17,1 | 14,8-19,3 | ns |
|  | 2014 | 35,4 | 31,3 – 39,5 | 36,8 | 34,0-39,6 | ns |
|  | 2021 | 8,8 | 6,4 – 11,2 | 13,9 | 12,-15,6 | ns |
| Access to expensive treatment should depend on the patient´s personal responsibility for the disease | 2008 | 18,5 | 15,2-21,7 | 19,1 | 16,7-21,4 | ns |
|  | 2014 | 41,0 | 36,9 – 45,1 | 42,8 | 39,9-45,6 | ns |
|  | 2021 | 18,7 | 15,3-22,0 | 22,0 | 20,0-24,0 | ns |
| Access to scarce organ transplants should depend on the patient´s personal responsibility for the disease | 2008 | 27,9 | 24,0-31,7 | 26,9 | 25,2-30,6 | ns |
|  | 2014 | 50,8 | 46,5-55,1 | 52,2 | 49,3-55,1 | ns |
|  | 2021 | 24,7 | 21,0-28,4 | 39,8 | 37,4-42,2 | * |
| Lower priority should be allotted to patients who violate a contract of changes in lifestyle | 2008 | 22,2 | 18,6-25,8 | 24,0 | 21,4-26,6 | ns |
|  | 2014 | 46,9 | 42,6-51,2 | 46,6 | 43,7-49,4 | ns |
|  | 2021 | 22,4 | 18,8-26,0 | 27,8 | 25,6-29,9 | ns |
| A patient who is responsible for the disease should pay additional co-payments | 2008 | 6,9 | 4,7-9,1 | 7,6 | 5,4-8,4 | ns |
|  | 2014 | 8,3 | 5,9-10,7 | 9,3 | 7,6-11,0 | ns |
|  | 2021 | 4,2 | 2,5-5,9 | 5,7 | 4,6-6,8 | ns |

***G Excerpt from the relevant part of the questionnaire***

1 Do you agree with the following statements?

(Alternative answers: Disagree completely, Disagree partly, Neutral, Agree partly, Agree completely)

– Healthcare priority should depend on the patient’s personal

responsibility for the disease

– Access to expensive treatment should depend on the

patient’s personal responsibility for the disease

– Access to scarce organ transplants should depend on the

patient’s personal responsibility for the disease

– Lower priority should be allotted to patients who violate

a contract of changes in lifestyle

– A patient who is responsible for the disease should pay

additional co-payments

2 Conditions where personal responsibility should influence the priority for health care:

(Alternative answers: Yes, No, Don’t know)

– Overweight/obesity

– Smoking

– Excessive alcohol consumption

– Drug abuse

– Lack of physical exercise

– High risk sports leading to injury/disease

– Poor quality nutrition

– Combination of the factors

– Violation of contract of changed life style
